# Acute profound sensorineural hearing loss as the initial manifestation of Hairy Cell Leukemia, Case Report and literature review

**DOI:** 10.1016/j.ijscr.2019.05.056

**Published:** 2019-07-09

**Authors:** Abdulaziz AlEnazi, Riyadh Alhedaithy, Abdulrhman Alfayez, Yazeed Alghonaim

**Affiliations:** a Department of Otorhinolaryngology - Head and Neck Surgery , Imam Abdulrahman Bin Faisal University, King Fahd Hospital of the University (KFHU), Al Khobar, Saudi Arabia; b Division of Otolaryngology – Head and Neck Surgery, Department of Surgery, King Abdulaziz Medical City, National Guard Health Affairs, Riyadh, Saudi Arabia; c King Saud Bin Abdulaziz University for Health Sciences, Division of Otolaryngology – Head and Neck Surgery, Department of Surgery, King Abdulaziz Medical City, National Guard Health Affairs, Riyadh, Saudi Arabia

**Keywords:** Hairy cell leukemia, Sudden hearing loss, Hematologic diseases, Case report

## Abstract

**Introduction:**

Hematologic diseases are rarely present with sudden hearing loss as an initial symptom. Although the precise cause of sudden sensorineural hearing loss has not been identified, several pathophysiological mechanisms have been proposed. However, a variety of hematologic diseases are among the causes of sudden onset deafness. This article represents the first reported case of Hairy cell leukemia (HCL) which presented with acute unilateral profound sensorineural hearing loss as an inital mainfestation.

**Presentation of case:**

A 41-year-old man presented with unilateral sudden hearing loss for one day’s duration was found to have HCL during a worked up for his hearing loss. The disease worsened, and there was no improvement in his hearing.

**Discussion:**

Sudden sensorineural hearing loss can rarely be seen as a paraneoplastic occurrence.Very few cases have been reported in the literature. It has been reported as a presenting symptom of leukemia in numerous clinical and histopathological reports concerning the hearing loss in hematologic diseases. Our case is an example of such a rare incidence.

**Conclusion:**

The purpose of this study is to draw physicians’ attention to the possible association between acute sensorineural hearing loss to HCL, and to highlights permanent deafness as a complication secondary to HCL. Furthermore, it is important to increase awareness on early diagnosis and treatment that may improve treatment outcomes.

## Highlights

• Hematologic diseases rarely present with sudden hearing lossas an initial symptom.
• There are few cases reported the association of hematology disorder with (SSNHL).
• This article represents the first reported case of Hairy cell leukemia (HCL), which presented with acute unilateral sudden profound sensorineural hearing loss (SSNHL) as an inital manifestation.

## 1 Introduction

Hairy cell leukemia (HCL) is a very heterogeneous group of mature lymphoid B-cell disorders, characterized by the identification of hairy cells, with a specific genetic profile, a different clinical course, and the need for appropriate treatment [[1], [2], [3]]. HCL, which is four to five times more frequent in men than women, and accounts for 2% of all leukemias with approximately 1000 new cases being reported in the United States each year [4], The association between leukemia and hearing loss was first recognized by Donne ´ [5] followed by Vidal [6] around the time in the mid-19th century. However, sudden sensorineural hearing loss (SSNHL) rarely involves Hematological disease as an initial manifestation. SSNHL has been described by De Kleyn in 1944 [7], and is defined as hearing loss of at least 30 dB in three sequential frequencies in the standard pure-tone audiogram over three days or less [8]. The reasons for sudden hearing loss in patients with a hematologic disorder, especially in leukemia, are to be reported related to leukemic infiltrate, hemorrhage, and of hyperviscosity blood [9], Typically, sudden hearing loss has no identiable etiology [10]. Nevertheless, it has been associated with malignancy secondary to carcinomatous or lymphomatous meningitis [11]. Sudden hearing loss has also been reported as a presenting symptom of chronic myeloid leukemia [12]. Proposed etiologies of sudden hearing loss include viral inflammation, vascular diseases, allergic reaction, rupture of intralabyrinthine membranes, and autoimmune diseases [13]. Detailed investigations can show a specific causes in about 10% of patients [14]. Therefore, investigation for such disorders should be performed in for all patients. Furthermore, there are few cases that have reported the association of hematologic disorder with SSNHL. This work has been reported in line with the SCARE criteria [15]. To the best of our knowledge, this is the first case of HCL with a sudden onset of unilateral sensorineural hearing loss as the initial presenting symptom. The purpose of this study is to draw attention to the possible association of acute sensorineural hearing loss as a complication secondary to HCL.

## 2 Presentation of case

**Fig. 1:**
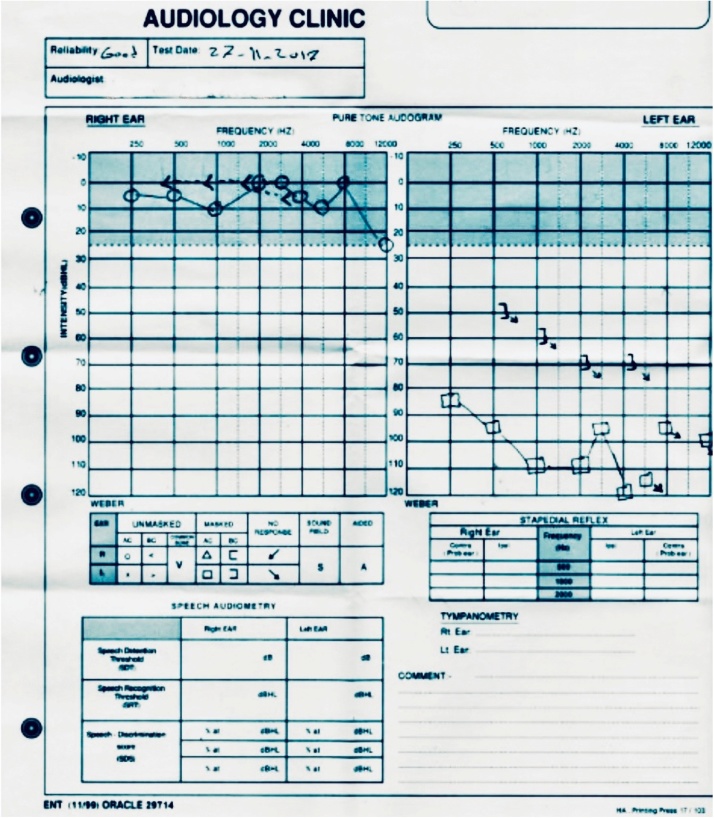
Pure tone audiometry with good Reliability revealed good hearing on her right ear but Sever to profound sensorineural hearing loss at mid and high frequencies on the left ear.

**Fig. 2:**
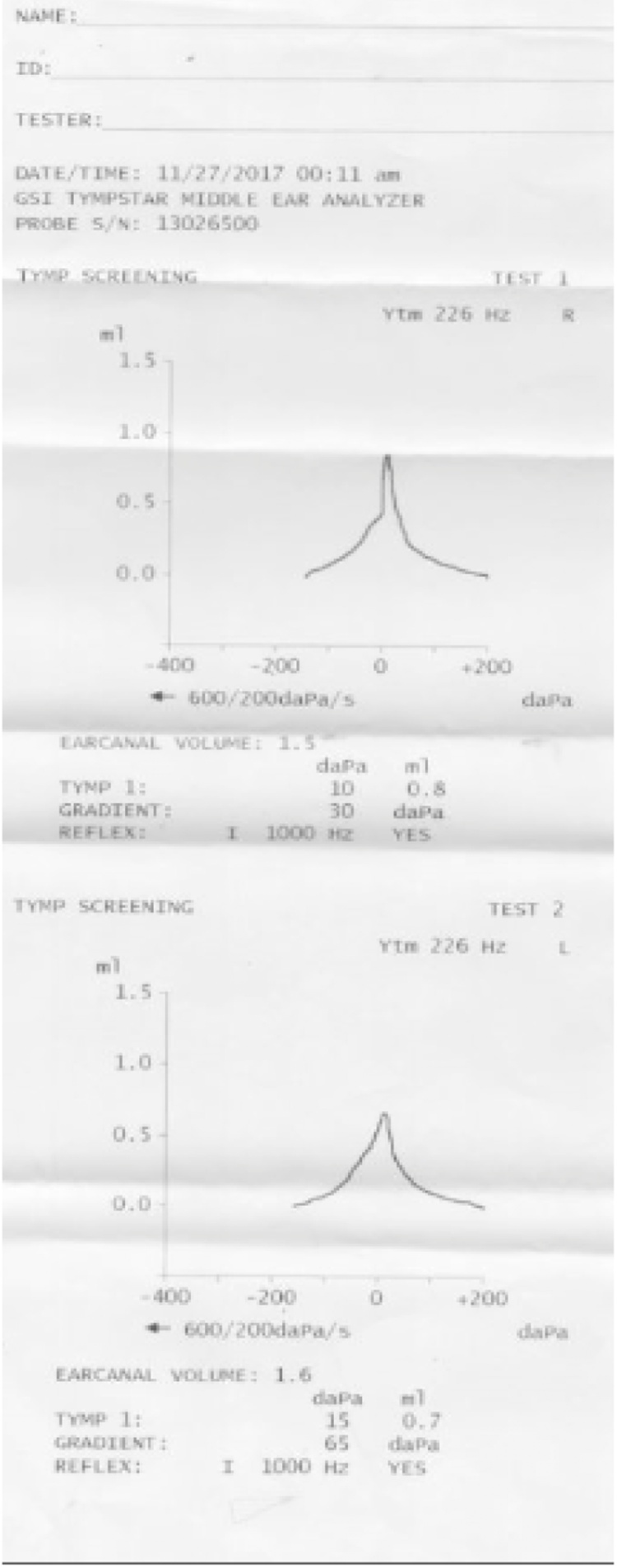
Type A Tympanogram of Both Ears with normal middle ear pressure.

A 41-year-old Egyptian man who moved to the Saudi Arabia approxmately 10 years ago presented with left hearing loss of sudden onset in the left ear for a duration of one day, accompanied by persistent non-pulsatile tinnitus, spinning sensation vertigo lasting for seconds which was aggravated by head movement to the left side, and fullness in the left ear for one day’s duration, along with abdominal pain and nausea. His past medical and surgical history were unremarkable except for bronchial asthma that was well controlled without medication. No history of ototoxic medication, trauma, recent infection, or family history was noted. His examination revealed vitally stable and not on distress, normal external auditory canals with intact tympanic membranes bilaterally. No nystagmus was detected. There was no lymph node enlargement in the neck, supraclavicular fossa, axillary, inguinal, or femoral regions. The spleen was enlarged on percussion. Cranial nerves were intact and motor strength was equal in all four extremities. Weber radiated to the right ear. Rinne + ve right side with no response on the affected side. The Pure tone audiometry revealed good hearing in her right ear but severe to profound sensorineural hearing loss at mid and high frequencies in the left ear (Fig. 1, Fig. 2). Other audiological and otoneurological examinations performed after admission revealed word discrimination scores of 100% in the right ear but 65% in the left ear. The patient was initially diagnosed with idiopathic sudden deafness, but upon further investigation he was diagnosed with leukemia. The patient was immediately transferred to the Hematology Department for the management. On initial impression the patient was considered to have been misdiagnosed at another hospital as (AML), he was started on 7 plus 3 regimen chemotherapy. Workup revealed a white blood cell count of WBC 6.28 × 10^9/L, with a marked increase in the granulocyte series, HGB 110 gm/l, MCV 105.2 Fl, PLT 162 × 10^9/L, Lymphocytes 1.63 × 10^9/L, and neutrophils 3.45 × 10^9/L., a blood smear test showed macrocytic red cells with mild anisopoikilocytosis. Few teardrop cells, spherocytes and rare nucleated red cells were seen. Platelets were unremarkable. Neutrophils were adequate but left shifted, and few of them showed minimal toxic granulation and Dohle bodies. Rare bare megakaryocytic nucleus was noted, which was a rare suspicious hairy cells were seen. A bone marrow aspirate from the right posterior iliac showed a 100-cell differential: of erythroid 50%, blasts 1%, promyelocytes 1%, myelocytes 16%, metamyelocytes 4%, band, segmented neutrophils 26%, and lymphocytes 2%. The morphology and flow were consistent with HCL. An immunohistochemistry evaluation showed a few scattered CD20 (clone L26) and positive B cells, which were estimated to be less than 5%. The bone was consistent with HCL. The bone marrow and flow cytometry were reviewed in our hospital and the Mayo clinic , where the diagnosis of HCL was confirmed the diagnosis of Hairy cell leukemia.

The patient was transfused with 18 unites of platelets. Radiological evaluation of the temporal bone and the brain, including computer tomography scan and magnetic resonance imaging revealed no particular abnormality, no cerebellopontine tumor or, any other pathology. The patient was treated with chemotherapy consisting of vincristine sulfate, daunorubicin hydrochloride, methotrexate, and prednisolone. Immediate treatment with the a higher dose of prednisone 60 mg taper courses of oral steroid was started. The patient was scheduled for an audiological follow up; however, but he was unable to tolerate the test and no subjective improvement of hearing was noted. However, an Intratympanic injection was not adminstered because the control of the disease was poor, and he subsequently developed severe thrombocytopenia, anal fistula and a rash. These complications aggravated his status, and further testing was difficult to perform.

## 3 Discussion

The precise cause of SSNHL has not been identified; however, several pathophysiological mechanisms have been proposed. The most common neoplastic disease associated with SSNHL is acoustic neuroma (vestibular schwannoma), which usually presents with unilateral tinnitus and progressive sensorineural hearing loss, although it can also present with sudden loss in a few cases [16]. SSNHL is rarely be seen as a paraneoplastic occurrence [17] and it has also been reported as a presenting symptom of chronic myeloid leukemia [18]. Numerous clinical histopathological studies have reported hearing loss in leukemia. Our case is an example of such a rare incidence. In our case, the type of leukemia underlying acute sensorineural hearing loss was HCL. However, the type of leukemia does not appear to be an important factor, since there are reports of different types of leukemia. Presenting acute sensorineural hearing loss as the initial manifestation [[19], [20], [21], [22]]. Furthermore, besides leukemia, other hematologic disorders and abnormal different conditions also appear to cause acute sensorineural hearing loss [24,25]. Bilateral involvement which was frequently observed in previous reports of leukemic patients complicated by sensorineural hearing loss has been frequently observed in previous reports of leukemic patients [[19], [20], [21], [22], [23]]. In our case, the hearing loss was unilateral which consistent with most cases in the literature, where bilateral involvement has been reported in less than 5% of the cases [26]. The aural symptoms such as Sensorineural hearing loss, tinnitus, and dizziness are not unusual.

Population studies of SSNHL show a wide age distribution, with an average of 50–60 years with no sex preference. Our patient was in his early forties age which makes this case different, due to different reasons such as the presence of leukemia. The evidence of SSNHL in the literature suggests: Vascular and Infectious causes. Furthermore, Studies have investigated several possible mechanisms, including atherosclerosis, hypotension, thrombophilia, vasospasm, hyperviscosity, and paradoxical embolism as risk factor [27]. Many viruses have been postulated to cause SNNHL [28]. However, in our case the patient was otherwise healthy with no significant medical history, and he didn't have a risk of Infectious, immune- mediated inner-ear disorder or ototoxicity causes of sudden sensorineural hearing loss. Such cases indicates the importance of differentiating between possible underlying diseases before arriving at diagnosis of diagnose idiopathic sudden deafness when we see a patient with sudden onset hearing loss of sudden onset. Unfortunately, in our case, an accurate diagnosis was possible only after the disease reached an advanced stage and was complicated by severe thrombocytopenia, anal fistula, a rash, and other symptoms. We regret that the patient did not have an earlier chance to be diagnosed properly before he developed sudden hearing loss.

## 4 Conclusion

This article represents a rare case in which acute sensorineural hearing loss occurred as an initial manifestation of HCL and not during the clinical course of the disease. To our knowledge, this is the onset sensorineural hearing loss as an initial-symptoms in HCL. The purpose of this study is to increase awareness regarding the development of acute sensorineural hearing secondary to HCL and the importance of early diagnosis and treatment, which may result in an improved hearing outcome. Hematological disorders such as HCL, should be considered to be among the causes of sudden onset deafness, despite the rarity of this presentation. In addition, further studies are also needed on this topic.

## Declaration of Competing Interest

There are no conflicts of interest.

## Funding

The author(s) received no specific funding for this work.

## Ethical approval

Case reports do not require ethical approval by our institution if no patient identification and case is anonymous.

## Consent

Written informed consent was obtained from the patient for publication of this case report and accompanying images. A copy of the written consent is available for review by the Editor-in-Chief of this journal upon request.

## Author contribution

Abdulaziz Alenzi: Study concept, writing the paper.

*Riyadh Alhedaithy,* Study concept and review the paper.

*Abdulrhman Alfayez* Study concept and review the paper.

*Yazeed Alghonaim* Study concept and review the paper.

## Registration of research studies

NA.

## Guarantor

Abdulaziz Saud Alenazi.

## Provenance and peer review

Not commissioned, externally peer-reviewed.
